# Preserving Genome Integrity: Unveiling the Roles of ESCRT Machinery

**DOI:** 10.3390/cells13151307

**Published:** 2024-08-05

**Authors:** Mattia La Torre, Romina Burla, Isabella Saggio

**Affiliations:** 1 Department of Biology and Biotechnologies “Charles Darwin”, Sapienza University, 00185 Rome, Italy; mattia.latorre@uniroma1.it (M.L.T.); romina.burla@uniroma1.it (R.B.); 2 CNR Institute of Molecular Biology and Pathology, 00185 Rome, Italy

**Keywords:** ESCRT, cell division, nuclear envelope sealing, midbody, genome integrity

## Abstract

The endosomal sorting complex required for transport (ESCRT) machinery is composed of an articulated architecture of proteins that assemble at multiple cellular sites. The ESCRT machinery is involved in pathways that are pivotal for the physiology of the cell, including vesicle transport, cell division, and membrane repair. The subunits of the ESCRT I complex are mainly responsible for anchoring the machinery to the action site. The ESCRT II subunits function to bridge and recruit the ESCRT III subunits. The latter are responsible for finalizing operations that, independently of the action site, involve the repair and fusion of membrane edges. In this review, we report on the data related to the activity of the ESCRT machinery at two sites: the nuclear membrane and the midbody and the bridge linking cells in the final stages of cytokinesis. In these contexts, the machinery plays a significant role for the protection of genome integrity by contributing to the control of the abscission checkpoint and to nuclear envelope reorganization and correlated resilience. Consistently, several studies show how the dysfunction of the ESCRT machinery causes genome damage and is a codriver of pathologies, such as laminopathies and cancer.

## 1. The ESCRT Machinery

### 1.1. The ESCRT Subunits

**Figure 1:**
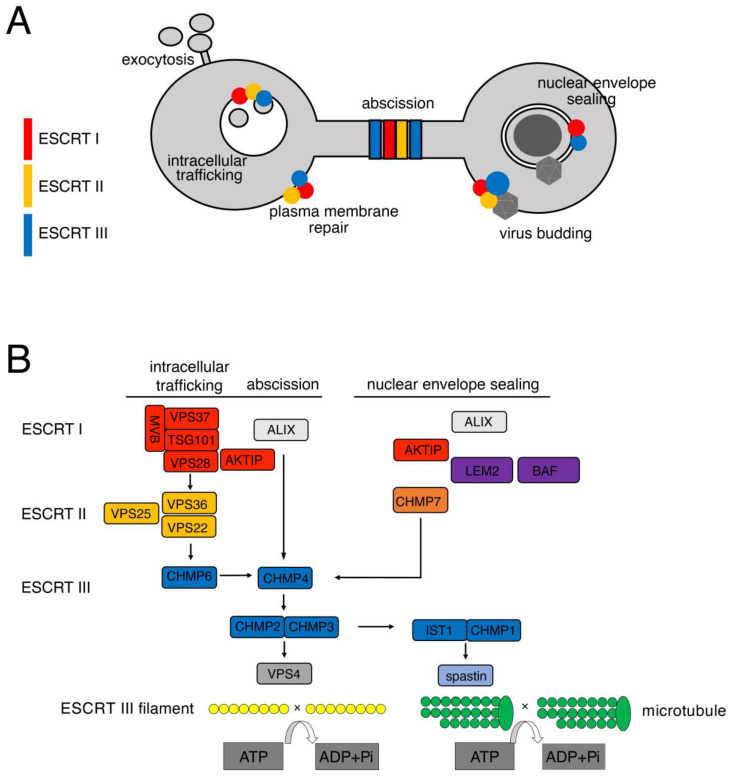
Cellular processes involving the ESCRT machinery. (**A**) Schematic representation of the functions of the ESCRT machinery. ESCRT I (red); ESCRT II (yellow); ESCRT III (blue), virus symbol (dark grey). (**B**) Schematic representation of the cascade of ESCRT complexes recruited at the site of action. ESCRT I (red); ESCRT II (yellow); ESCRT III (blue).

The endosomal sorting complex required for transport (ESCRT) is a machinery composed of protein complexes that contribute to multiple cellular processes, such as cytokinesis [1], endosome maturation [2], neuronal pruning [3,4], the nuclear envelope [5,6], and plasma membrane repair [7,8,9]. In addition, the ESCRT machinery has been implicated in viral replication and budding [10,11,12,13] (Figure 1A). The complexes or groups of the machinery are the ESCRT 0, ESCRT I, ESCRT II, and ESCRT III (Figure 1B). By parallel mechanisms, operated at different sites, the ESCRT 0 and ESCRT I subunits initiate the process mediated by the ESCRT machinery and recruit the ESCRT II complex. The ESCRT II subunits help in the recruitment and assembly of the ESCRT III complex. Eventually, the ESCRT III subunits mediate inverse membrane involution finalizing membrane scission or sealing [14,15,16,17,18].

The definition and functional characterization of the different components of the ESCRT machinery were first assessed in yeast and successively paralleled in mammals and other organisms (Table 1). Yeast ESCRT subunits include the ESCRT 0 Vps27, corresponding to HRS-HGS in mammals [19,20]; the ESCRT I Vps23, Vps28, Vps37, and Mvb12 corresponding to, respectively, VPS23 or TSG101 [21], VPS28 [22], VPS37a, b, c, d [23,24], and MVB12a, b in mammals [15,25]; the ESCRT II Vps36, Vps22, and Vps25 corresponding to mammalian EAP45, EAP30, and EAP20, respectively [17,18,26,27,28]; and the ESCRT III Vps2, Vps24, Snf7, and Vps20, corresponding to mammalian CHMP2A, B, CHMP3, CHMP4A, B, C, and CHMP6, respectively [18,29,30,31,32,33]. CHMP7, which works on nuclear envelope sealing in mammals, has been described as a hybrid ESCRT II/III subunit [32,34,35,36]. In addition to the ESCRT subunits, several accessory proteins contribute to the activity of the machinery. These include Bro1, in yeast, corresponding to ALIX in mammals [37], and IST1 that works in concert with the ESCRT III complex and possesses structural similarities to the ESCRT III CHMP3 [38]. All the ESCRT components, except the ESCRT III subunits, bind cargo and/or other ESCRT components. The ESCRT III subunits serve to complete the pathway operated by the machinery and for its disassembly, which happens via the activity of the AAA + ATPase VPS4 complex [18,33,39].

The comprehension of the full picture of the structure–function organization of the ESCRT subunits and of their site of action is in continuous evolution and expansion, along with the identification of new ESCRT-associated factors. AKTIP, for example, is a recently discovered ESCRT I associated protein. The database annotation of AKTIP points to human TSG101 as the AKTIP top-hit homologue with high probability. AKTIP, as the ESCRT I VPS23/TSG101, includes the ubiquitin E2 variant domain and interacts with the ESCRT I VPS28 [71]. Differently from TSG101, AKTIP does not include a proline-rich domain required for the interaction with CEP55 [1,71]. AKTIP is detected in the nucleus and in the cytoplasm and is enriched in distinct foci at the nuclear rim [72,73,74,75]. AKTIP has been associated with HOOKs, a group of proteins impinging on vesicle trafficking, and with ESCRT components in cytokinesis [71,76]. During cytokinesis, AKTIP is recruited at the midbody together with the ESCRT subunits.

### 1.2. Structural Organization of the ESCRT Subunits

In support of its pivotal function in the biology of the cell, the ESCRT machinery has ancient origins. In fact, many archaeal species possess ESCRT proteins, and the machinery is conserved during evolution. Metagenome analyses show that Asgard archaea, for example, possess components of the ESCRT I, ESCRT II, and ESCRT III complexes, and, as in mammals, ESCRT III subunits execute the final stages of membrane processing [77,78,79]. Multiple studies of reverse genetics highlight the presence of ESCRT I, ESCRT II, and ESCRT III genes in *Drosophila melanogaster* [46,47,80]. Recent evidence describes new functions for *Drosophila* ESCRT subunits, such as that of the ESCRT III component Shrub that maintains the septate junction and guarantees epithelial tissue integrity in larvae [81].

**Figure 2:**
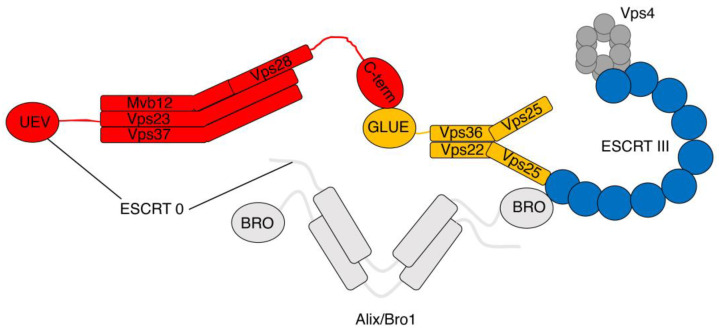
Structure of the ESCRT complexes. Schematic representation of the structural organization of the ESCRT complexes. The UEV domain of Vps23 is responsible for the interaction with ESCRT 0 components (black line), whereas the C-terminal domain of Vps28 interacts with the GLUE domain of Vps36. The Y shaped ESCRT II complex is responsible for the recruitment of ESCRT III subunits. Vps4 is recruited by ESCRT III subunits. ESCRT I (red); ESCRT II (yellow); ESCRT III (blue).

Structural studies have shed light on the ESCRT protein domains that are involved in the assembly of the different complexes (Figure 2). In the ESCRT I complex, the core is made of helical hairpins from the three components: Vps23, Vps28, and Vps37 [16]. This core tethers the ubiquitin E2-variant domain of Vsp23 to the ESCRT I C-terminal domain of Vps28 [44,82]. According to the original structural model [19], ESCRT II subunits contain tandem repeats of winged-helix domains and are recruited by ESCRT I via the so-called GLUE of the ESCRT II Vps36 [17,83]. ESCRT III subunits do not form stable complexes, and attempts to perform structural studies were slowed by this aspect. However, the crystal structure of human VPS24, CHMP3, was revealed showing that it includes five helices with a core of a hairpin formed by two of the helices [38,84].

In the following paragraphs, we review the data concerning the role of the different subunits of the ESCRT machinery in controlling the integrity of the nuclear envelope on the one side and the process of abscission on the other. In both cases, we analyze how the machinery impacts the integrity of the genome.

## 2. The ESCRT Machinery and Genome Integrity at the Nuclear Envelope

### 2.1. The Organization of the Interphase Nuclear Envelope

In eukaryotic cells, the genome is separated from the nucleoplasm by the nuclear envelope. This is composed of the outer nuclear membrane in continuation with the endoplasmic reticulum and by the inner nuclear membrane juxtaposed to the lamina. This latter element is a meshwork composed mainly of lamin type A and B [85,86,87,88]. Lamins type A are encoded by the LMNA gene and have two isoforms, A and C, produced by alternative splicing events. B-type lamins are encoded by the LMNB1 and LMNB2 genes, respectively. Most metazoans express B-type lamins in support of a critical and conserved role of the lamina in the organization of the nuclear organelle. B-type lamins are essential and expressed during development; A-type lamins are present only in differentiated cells. B-type lamins are mostly detected at the nuclear envelope. A-type lamins are detected also in the nucleoplasm serving in multiple roles, including the control of chromatin organization and function [89,90,91]. The nuclear envelope is interrupted by the nuclear pore complexes to which lamins provide support. Super-resolution microscopy analyses have shown that the nuclear pore complex component TPR is a determinant in the association of the nuclear pore complex to lamin C [92,93,94].

The lamina is also the resident site for a plethora of proteins. Smoyer et al. identified more than 400 inner nuclear membrane proteins [95]. Among these, there is the LAP2-Emerin-Man1 (LEM)-domain protein subgroup that includes MAN1 [96], LEM2 [97], the lamina-associated polypeptide 2 (LAP2) [98], and emerin [99,100,101]. Other well characterized proteins involved in the integrity and function of the nuclear envelope are the SUN-domain proteins, SUN1 [102] and SUN2 [103,104], and the lamin B receptor [105,106,107]. In addition, the inner nuclear membrane is in contact with the cytoplasm via the LINC (Linkers of the nucleoskeleton to the cytoskeleton) protein complexes [108,109]. LINC factors associate with the lamina or with lamin associated proteins and traverse the outer nuclear membrane to reach at the cytoplasmic side [110,111].

The nuclear envelope, the lamina, and the lamin-interacting proteins contribute to the spatial distribution of chromatin. Chromatin structural compartmentalization was first described by Carl Rabl and Theodor Boveri and refined by the seminal works of Cremer et al. [112] and Cremer et al. [113]. More recent studies have given the molecular details of the spatial architecture of the genome inside the nucleus based mostly on the usage of chromatin conformation capture, 3C techniques [114,115]. Integrating the seminal studies defining the compartmentalization of chromosomes in discrete territories with 3C-technique based data allowed the identification of chromatin topologically associating domains (TADs) [116]. TADs are genomic stretches stabilized by the presence of transcription factors and cohesins [117,118]. 3C techniques have also been useful to define the presence and molecular characteristics of genomic stretches interacting with lamins, the lamin associating domains (LADs) [119,120,121]. Immunofluorescence and biochemical analyses have, in addition, shown how the nuclear envelope associates with the chromatin via the LEM domain proteins [101,122,123]. MAN1 and emerin bridge with the chromatin via another factor named BAF [124,125,126]. The lamin B receptor contacts chromatin through HP1 [127,128,129].

### 2.2. The Dynamics of the Nuclear Envelope and Role of ESCRTs

The process of cell division exhibits variations across different organisms and cell types. In metazoans and higher eukaryotes, open mitosis is prevalent. This type of mitosis is characterized by the fragmentation of the nuclear envelope. In contrast, lower eukaryotes like S. cerevisiae and S pombe commonly undergo closed mitosis, where the nuclear envelope remains intact [130,131]. Notably, exceptions exist, as seen in *Cryptococcus neoformans* and certain strains of *Ustilago*, which display a unique form of open mitosis. Some higher eukaryotes engage in semi-open mitosis, where the rearrangements of the nuclear envelope are minimal [132,133,134].

In open mitosis, the breakdown of the nuclear envelope requires a series of intricate events leading to the temporary disassembly of the nuclear envelope that then has to be followed by its reformation [135]. This is a carefully regulated process initiated during prophase [136]. Several molecular mechanisms contribute to this event. Notably, the phosphorylation-mediated disassembly of the nuclear pore complex marks a critical step. Kinases such as CDK1, NEK, and PLK1 phosphorylate nucleoporins, leading to nuclear pore complex disintegration [136]. Concurrently, lamins undergo phosphorylation by kinases like CDK1/cyclin B, initiating their depolymerization and favoring subsequent events in nuclear envelope breakdown [135,137,138]. Spindle microtubule-generated forces contribute to nuclear envelope retraction, creating tension that results in the stretching and tearing of the nuclear envelope, ultimately leading to its fragmentation [139,140]. Dynein is needed for attaching spindle microtubules to the nuclear envelope, creating pulling forces towards the centrosome [141,142,143,144]. The endoplasmic reticulum undergoes significant remodeling during G2/M transition, further contributing to the completion of nuclear envelope breakdown [145,146,147]. Finally, the orchestration of nuclear envelope breakdown involves the phosphorylation of nuclear envelope-associated proteins, which disrupts protein–protein interactions and triggers the dissociation of these components contributing to the overall structural rearrangement of the nuclear envelope [148,149,150,151,152,153]. The retraction of the nuclear envelope facilitated by mitotic spindle microtubules involves the withdrawal of the nuclear envelope from chromatin, which is an actively regulated process [133,154,155]. Studies in different organisms, including fission yeast, demonstrate the active regulation of chromatin detachment through post-translational modifications and the involvement of the protein complex Lem2-Nur1 [156,157].

**Figure 3:**
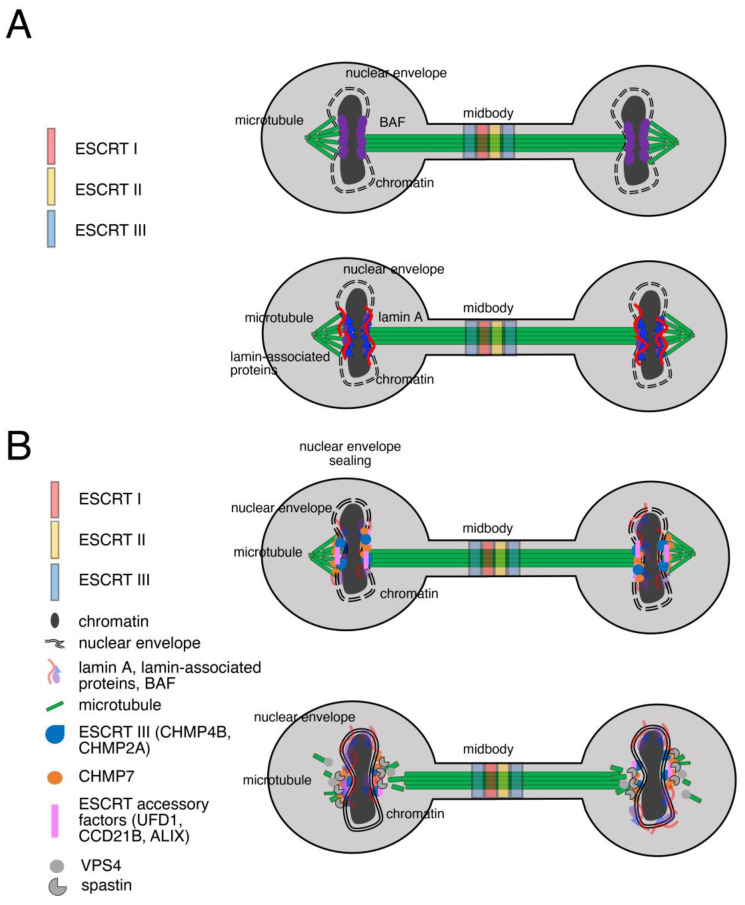
ESCRT recruitment and function at the nuclear envelope. (**A**) Schematic representation of the recruitment of the core proteins at the chromatin at the end of mitosis. ESCRT I (red); ESCRT II (yellow); ESCRT III (blue); BAF (purple); chromatin (dark grey); microtubule (green); lamin A (red curved line); lamin-associated proteins (blue triangle); nuclear envelope (black double dotted line). (**B**) Schematic representation of the recruitment of the ESCRT subunits during nuclear envelope sealing. ESCRT I (red); ESCRT II (yellow); ESCRT III (blue).

Following nuclear envelope breakdown, when mitosis is not yet completed, the nucleus starts to reorganize its architecture for the next interphase. Here, a set of proteins is recruited progressively around the chromatin, constituting the so-called core region (Figure 3A). BAF is first detected at the chromatin, followed by multiple lamin-associated proteins, as LEM2 and LAP2alpha, followed by emerin, LAP2beta, and MAN1 along with lamin A [158,159,160,161]. During telophase, the organization of the two daughter rims around chromatin is visible along with the midbody region between the nascent cells. In mammalian cells, telomeres have a defined dynamic in the anaphase to telophase stage, during which they are enriched at the nuclear envelope through interactions between SUN1 and the telomeric protein RAP1. This distribution of telomeres is presumed to contribute to chromatin domain reorganization including the juxtaposition of heterochromatin at the nuclear lamina [162,163].

To complete the compartmentalization of the genome at the end of the mitotic process, the nuclear envelope discontinuities, due also to the presence of residual microtubules traversing the nascent rim, are repaired by the ESCRT machinery (Figure 3B). The subunits involved in this process are the ESCRT III CHMP4B and CHMP2A and the specialized nuclear ESCRT II/III hybrid factor CHMP7, along with the ESCRT accessory factors UFD1, CCD21B, and ALIX [5,6,36,164,165,166,167]. VPS4 and spastin complete the process of nuclear envelope sealing by regulating the disassembly of the complexes, acting, respectively, on the ESCRT III and on microtubules [5,157,168].

An interesting link has been established between CHMP7 and LEM2, which relates as well to the spatiotemporal interpretation of the nuclear reassembly process. Namely, liquid–liquid phase separation has been suggested for the assembly of LEM2 and CHMP7 around residual spindle microtubule bundles, in connection with the chromatin-binding factor BAF [35,167,169,170,171,172]. LEM2 is thus reputed to be a transmembrane ESCRT adaptor protein, and this vision highlights that the nuclear reassembly dynamic phase involves chromatin, chromatin-binding factors, lamin-binding factors, the ESCRT machinery, and, eventually, lamin [35,169].

The activity of the ESCRT III in repairing nuclear envelope discontinuities is required also during the interphase. Indeed, nuclear rim ruptures occur in the interphase during cell migration in confined space, upon mechanical stress, in cancer metastases or consequent to genetic mutations [173,174,175,176]. These nuclear ruptures are repaired via the concerted action of BAF, LEM2, and ESCRT III. Specifically, cytoplasmic BAF localizes onto DNA at nuclear ruptures, contributing to the recruitment of LEM2 and CHMP7 [177].

### 2.3. Nuclear ESCRT Genome Integrity

The massive process of nuclear reorganization, happening in the final phases of mitosis, calls for an interdependence between the correct reorganization of chromatin in the daughter cells and the activity of the ESCRT machinery. Chromothripsis has been associated with nuclear envelope composition defects and defective nuclear pore complex assembly, impacting genome integrity and function [178]. In analogy, in micronuclei, a defective rim composition has been related with the control of ESCRT III recruitment and function. Indeed, although the ESCRT III subunit CHMP7 is correctly recruited at the micronuclear rim, its spatiotemporal distribution is not correctly restricted [179]. This dysfunctionality is a driver of membrane deformation and DNA damage [180,181]. Vietri and co-workers suggest that the ESCRT III machinery is a “double-edged sword”, driving repair and compartmentalization in wildtype conditions but performing as a damaging agent in dysfunctional conditions [179]. ESCRT III subunits have been also implicated in the regulation of nuclear envelope channels, which contribute to the reintegration of chromosome fragments into the nuclei, impacting genome integrity [182,183]. Another aspect of the role of the ESCRT machinery in nuclear envelope dynamics is also suggested by a study focusing on nuclear invaginations. Here, using C. elegans as a model system, the authors demonstrate how, in the early phase of organismal development, the ESCRT machinery contributes to nuclear membrane remodeling and to the preservation of genome integrity [184].

Two seminal papers have described the role of the ESCRT machinery in preserving the intertwined integrity of the nuclear rim in the interphase and that of the genome. Denais et al. and Raab et al. showed that mechanically stressed nuclei lose their circularity and display nuclear blebs, where the ESCRT III subunits accumulate [7,8]. These studies showed that ESCRT III-mediated repair is needed for preserving the genome from DNA damage and opened the route to an area of research focusing on the direct mechanistic relationship between the ESCRT machinery and genome fragility in a clinical perspective as well.

### 2.4. Nuclear ESCRTs and Disease

The role of ESCRT subunits as guardians of nuclear envelope integrity inevitably ties their dysfunction to pathological situations affecting the nucleus and its genomic content. Failed nuclear envelope repair in the interphase after rupture or in the terminal phase of mitosis leads, as described, to altered genome organization, DNA migration out of the nucleus, and DNA exposure to enzymes that alter if not prevent its function, which induces the pathological state of the cell and of the organism.

From a mechanical perspective, the fragility of the nuclear membrane and/or the disorganization of chromatin decrease the nuclear and cellular resilience to stress [185,186]. This latter aspect becomes particularly relevant in the context of pathologies characterized by fragile nuclei. An example is Hutchison Gilford Progeria Syndrome, which is linked to a mutation in the LMNA gene, which leads to the production of a truncated, aberrant, and not properly matured form of lamin A that phenotypically generates deformed nuclei displaying surface blebs, a disorganized genome, and an altered distribution of nuclear proteins dependent on the lamin meshwork. It is not surprising that, in this context, the modulation of ESCRT subunits impact the phenotype [187].

Moreover, the role exerted by the ESCRT machinery at the nuclear envelope impacts tumor aggressiveness in the metastasis process (reviewed in [188,189]). In fact, when tumor cells undergo migration through tight interstitial spaces within tissues, they necessitate the significant deformation of both the cell and its nucleus as also shown by Denais et al. in studies investigating mammalian tumor cell migration within confined microenvironments [8].

## 3. The ESCRT Machinery and Genome Integrity at the Midbody

### 3.1. Cell Abscission and ESCRT Complexes

**Figure 4:**
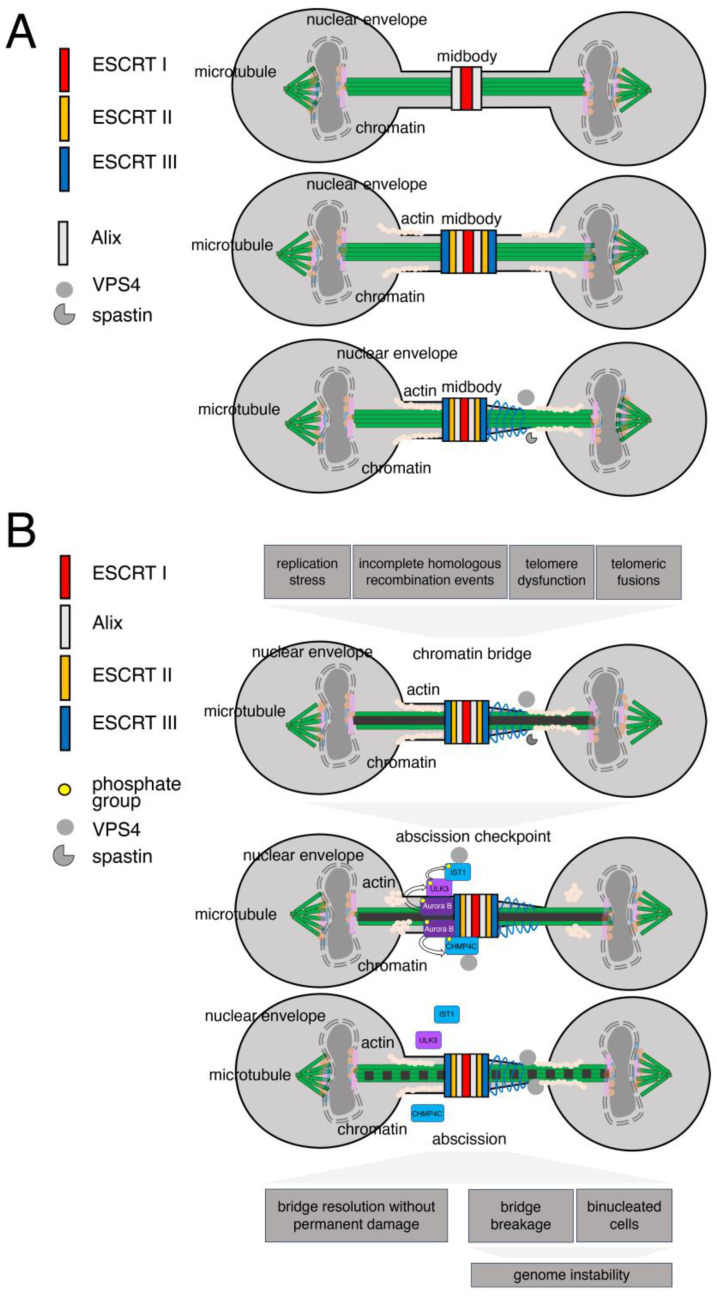
ESCRT recruitment and function in abscission. (**A**) Schematic of the midbody during abscission in which ESCRT I (red) and ALIX (light grey) are recruited at the middle of the tubulin (green) bridge by central spindlin and CEP55. ESCRT I and ALIX recruit ESCRT III subunits (blue). In the final stage of abscission, the ESCRT III subunits form spirals (blue spirals) and recruit spastin, which trims the microtubules, and VPS24. (**B**) Schematic representation of the abscission checkpoint activation triggered by the presence of a chromatin bridge (dark grey line). Phosphorylation (curved arrow); proteins recruited at core region of chromatin (light pink rectangle, light orange circle, light blue rectangle); actin (white circles in line and organized in patches).

Cytokinesis is a multistep process that permits the correct physical separation of daughter cells following nuclear division. It includes the assembly of the actomyosin contractile actin ring to achieve a primary constriction leading to the formation of the intercellular bridge between the two daughter cells, the physical reorganization of microtubules during bridge formation, and the secondary constriction of the intercellular bridge, ending with the final abscission step [190] (Figure 4A). The ESCRT machinery functions at the heart of cell abscission and orchestrates membrane fission events. It operates in abscission via the sequential assembly of ESCRT I, II, and III subunits at the midbody, the central region of the intercellular bridge that links the daughter cells prior to their separation. The process of ESCRT assembly at this site is initiated by the central spindlin subunit MKLP1 and by CEP55 [191,192]. CEP55 is responsible for the recruitment of the ESCRT I component TSG101 and of the accessory ESCRT ALIX [1,10,193]. The ESCRT I component TSG101 is found at the midbody in association with septins and with AKTIP [71,194]. Recent studies in CEP55-knockout mice have shown that ESCRT recruitment at the midbody can occur also via CEP55-independent mechanisms [195]. Along the same line, Drosophila has no CEP55, and ESCRT recruitment to the midbody is mediated by the human MKLP1 orthologue [70].

The microscopical analysis at a 100 to 200 nanometer resolution scale has permitted the visualization of the ESCRT super-structures, which form at the midbody and evolve through the different phases of abscission [196,197,198]. In the early phase, the ESCRT I and II subunits form packed circular structures at the center of the midbody [194]. ALIX and ESCRT II subunits form double rings next to the central midbody [199]. ESCRT III subunits, including CHMP2A, CHMP4B, and IST1, form double rings at the two sides of the ESCRT I/II structures [200,201]. The TSG101 homologue AKTIP locates at the midbody, forming a ring in the central zone of the bridge, in close association with TSG101 and in proximity to ESCRT III subunits [71]. In the late phase of abscission, the ESCRT III rings are transformed into spirals leading to the completeness of cell division [196,200,202]. This latter process depends on the ATPase VPS4 [39,203]. In this final phase, CHMP1B-dependent recruitment of the ATPase spastin occurs to finalize the intercellular bridge microtubule severing [62,63,64,65,66,67,68,69,70,71,72,73,74,75,76,77,78,79,80,81,82,83,84,85,86,87,88,89,90,91,92,93,94,95,96,97,98,99,100,101,102,103,104,105,106,107,108,109,110,111,112,113,114,115,116,117,118,119,120,121,122,123,124,125,126,127,128,129,130,131,132,133,134,135,136,137,138,139,140,141,142,143,144,145,146,147,148,149,150,151,152,153,154,155,156,157,158,159,160,161,162,163,164,165,166,167,168,169,170,171,172,173,174,175,176,177,178,179,180,181,182,183,184,185,186,187,188,189,190,191,192,193,194,195,196,197,198,199,200,201,202,203,204].

### 3.2. Abscission Check Point and Chromosome Integrity

The completion of abscission must be intimately coordinated with the correct chromosome distribution into the daughter cells, which preestablishes an interlink between chromosome and genome integrity on one side and the activity of the ESCRT machinery at the intercellular bridge on the other. Actually, chromosome segregation and ESCRT activity during abscission are commonly controlled by a checkpoint [205,206]. This abscission checkpoint is present in budding yeast (NoCut) and involves the kinase IPL1/Aurora [207,208]. In human cells, the kinase Aurora B controls the checkpoint guiding the localization and function of ATPases at the midbody [209]. Aurora B localizes to the midbody in the telophase inside the central region (or Flemming body) [209,210], where it targets CHMP4C and VPS4 [211,212,213]. Consistently, when the expression of Aurora B is reduced, the localization of the ESCRT III CHMP4C is altered [214].

The abscission checkpoint is driven by stresses, among which the most studied is the presence of anaphase chromatin bridges in association with chromosome integrity [215]. Stretches of DNA linking the two daughter cell genomes can be driven by replication defects, by incomplete homologous recombination events, or by telomere dysfunction and telomeric fusions [216,217,218,219,220]. DNA bridges are divided into ordinary and ultrafine. The latter are detected exclusively by staining the associated proteins, while ordinary DNA bridges are visible with conventional DNA staining methods [221,222]. Aurora B localizes at the midbody in response to the presence of these chromatin bridges (Figure 4B). Its recruitment is controlled by the Mre11-Rad50-Nbs1 (MRN) complex, the DNA double-strand break signaling kinase ATM, and its target CHK2 [223]. To revert Aurora B activity and overcome the abscission checkpoint, specialized factors localize at the midbody as RIF1 and PP1γ and PKCε [214,224,225]. The data suggest that RIF1 and PP1γ counteract Aurora B dependent phosphorylation of the ESCRT subunit CHMP4C [224]. The kinase ULK3, whose ESCRT target is IST1, also localizes at the midbody and controls abscission [226].

In parallel, to protect and stabilize chromosome bridges, cells reduce the depolymerization of actin filaments at the bridge and produce actin patches at either side of the bridge [227]. These latter structures could contribute to reducing the tension at the bridges by counteracting the velocity by which the daughter cells move when separating from each other [228]. Importantly, actin delays recruitment of ESCRT III proteins at the abscission site [229].

The abscission checkpoint bodies, consisting of cytoplasmic elements containing phosphorylated Aurora B, CHMP4C, CHMP4B, and ALIX, are a further element in the abscission picture, whose mechanistic role is yet to be fully unraveled [230].

Notwithstanding the abundant control of the abscission timing and the protection of chromatin bridges, the outcome of cytokinesis in the presence of these structures can be detrimental for genome integrity (Figure 4B). While in the best-case scenario stable chromatin bridges can be resolved without permanent DNA damage, unstable bridges and abscission control defects can lead to chromatin breakage, DNA damage, breakage–fusion–bridge cycles, and kataegis and chromothripsis [219,231,232]. In this latter process, clusters of localized rearrangements are randomly reassembled by DNA repair or aberrant DNA replication generating profound genome alterations [233]. Chromatin bridges can also lead to cleavage furrow regression, tetraploid cells, and chromosomal instability [234,235]. The cytoplasmic exonuclease TREX1 has also been implicated in cleaving chromosome bridges [219,236]. Finally, the presence of chromatin bridges also drives the production of micronuclei containing bridge DNA [237,238,239].

### 3.3. Abscission Defects and Cancer

The precise determinants of the destiny of chromosomal bridges are yet to be unraveled. It is not yet fully understood what drives chromatin bridge breakage or furrow regression. In either case, the presence of chromatin bridges and abscission checkpoint defects are drivers of genomic alterations and chromosome instability and are associated with cancer development and/or aggressiveness [240,241,242]. This highlights the importance of exploring the properties of ESCRT subunits for the understanding of new molecular cascades driving cancer and to identify new therapeutic targets. Several studies have already given insights into this perspective. The increase in Aurora B expression, for example, has been described in several tumor types and is associated with unfavorable prognosis [243,244,245]. Moreover, a CHMP4C polymorphism impairing ALIX-binding activity, has been associated with ovarian cancer [246,247].

## 4. Conclusions

Both for its role in the abscission process and in safeguarding the integrity of the nuclear membrane of mitotic and interphase cells, the ESCRT machinery profoundly impacts the correct organization of the genome and the dysfunction that ensues. By directly repairing breaches in the nuclear membrane, by controlling the abscission process, and, indirectly, by influencing genome organization, the machinery has a strong impact on cellular resilience and organismal pathologies. For future studies in the field, it will be interesting to reflect on the continuum of events linking the final stages of nuclear membrane organization and the cleavage of the bridge linking nuclei exiting division. It is possible that with a dynamic analysis of these events, enabled by modern cytological analysis tools, new interpretations can be offered.

Finally, an aspect deserving further study is the potential identification of individual ESCRT components as therapeutic targets or tools. This can be achieved either by targeting synthetic lethal dependencies, as already demonstrated for VPS4, or by correcting expression defects [248,249]. In either case, the genes themselves or molecules that control/mimic their expression can provide new therapeutic avenues to explore in various pathological contexts.

## Figures and Tables

**Table 1:** ESCRT components in yeast, flies, and mammals.

Complex Name	Yeast (*S. cerevisiae*)	Flies (*D. melanogaster*)	Mammals
ESCRT 0	Vps27 [40]	Hrs [41]	HRS-HGS [20]
	Hse1 [40]	dmel/stam [42]	STAM1, 2 [43]
ESCRT 1	Vps23 [44]	erupted/tsg101 [45]	TSG101 [21]
	Vps28 [44]	dvps28 [46,47]	VPS28 [22]
	Vps37 [44]	vps37A *, vps37B [48]	VPS37a [23]
			VPS37b [23]
			VPS37c [24]
			VPS37d [23]
	Mvb12 [44]	mvb12 [49]	MVB12a [15,25]
			MVB12b [15,25]
ESCRT II	Vps36 [17]	vps36 [50,51]	EAP45 (VPS36) [26,27,28]
	Vps25 [17]	vps25 [47,52]	EAP20 (VPS25) [26]
	Vps22 [17]	larsen/vps22 [50,53]	EAP30 (VPS22) [26]
ESCRT II/III	Chm7 [54,55]	CG5498 *	CHMP7 [34,35,36]
ESCRT III and associated proteins	Vps2 [29]	vps2 [56]	CHMP2A [29]
			CHMP2B [29]
	Vps24 [29]	vps24 [47]	CHMP3 [29]
	Snf7 [29]	shrub [47,57]	CHMP4A [30]
			CHMP4B [30]
			CHMP4C [30]
	Vps20 [29]	vps20 [58]	CHMP6 [31]
	Ist1 [59]	ist1 [60,61]	IST1 [38]
	Vps60 [29]	chmp5 [60,61]	CHMP5 [61]
	Did2 [29]	chmp1 [61]	CHMP1A [62,63]
			CHMP1B [62,63]
VPS4-ATPase complex	Vps4 [64]	vps4 [3,65]	VPS4A [32,33]
			VPS4B [32,33]
	Vta1 [66]	vta1 * [67]	LIP5 (VTA1) [68]
BRO1 proteins	Bro1 [69]	ALiX (CG12876) [60,70]	ALIX (PDCD6IP) [37]

* predicted ortholog (Flybase).
